# Prospecting major genes in dairy buffaloes

**DOI:** 10.1186/s12864-015-1986-2

**Published:** 2015-10-28

**Authors:** GMF de Camargo, RR Aspilcueta-Borquis, MRS Fortes, R. Porto-Neto, DF Cardoso, DJA Santos, SA Lehnert, A. Reverter, SS Moore, H. Tonhati

**Affiliations:** Universidade Estadual Paulista (Unesp), Faculdade de Ciências Agrárias e Veterinárias, Departamento de Zootecnia, Via de acesso Professor Paulo Donato Castelane, Jaboticabal, SP 14884-900 Brazil; School of Chemistry and Molecular Bioscience, The University of Queensland, St Lucia, Brisbane, QLD 4072 Australia; Commonwealth Scientific and Industrial Research Organization, Agriculture Flagship, St Lucia, Brisbane, QLD 4072 Australia; Queensland Alliance for Agriculture and Food Innovation, Centre for Animal Science, The University of Queensland, Brisbane, QLD 4067 Australia

**Keywords:** *Bubalus bubalis*, SNP, Reproduction, Milk, Candidate genes, GWAS

## Abstract

**Background:**

Asian buffaloes (*Bubalus bubalis*) have an important socio-economic role. The majority of the population is situated in developing countries. Due to the scarce resources in these countries, very few species-specific biotechnology tools exist and a lot of cattle-derived technologies are applied to buffaloes. However, the application of cattle genomic tools to buffaloes is not straightforward and, as results suggested, despite genome sequences similarity the genetic polymorphisms are different.

**Results:**

The first SNP chip genotyping platform designed specifically for buffaloes has recently become available. Herein, a genome-wide association study (GWAS) and gene network analysis carried out in buffaloes is presented. Target phenotypes were six milk production and four reproductive traits. GWAS identified SNP with significant associations and suggested candidate genes that were specific to each trait and also genes with pleiotropic effect, associated to multiple traits.

**Conclusions:**

Network predictions of interactions between these candidate genes may guide further molecular analyses in search of disruptive mutations, help select genes for functional experiments and evidence metabolism differences in comparison to cattle. The cattle SNP chip does not offer an optimal coverage of buffalo genome, thereafter the development of new buffalo-specific genetic technologies is warranted. An annotated reference genome would greatly facilitate genetic research, with potential impact to buffalo-based dairy production.

**Electronic supplementary material:**

The online version of this article (doi:10.1186/s12864-015-1986-2) contains supplementary material, which is available to authorized users.

## Background

Asian buffaloes are a livestock species with a high socio-economic importance and promising characteristics for production. The species is mostly found in developing countries integrating production system by providing meat and milk to local communities. Asian buffaloes are also used as draught animals. In developed countries, such as Italy, the buffalo population is selected for dairy production, specially the production of mozzarella cheese, the most famous trademark product. Buffalo milk has high fat content and solids concentration and these intrinsic characteristics are favourable for cheese manufacturing.

There are two species of domesticated buffaloes: river or water buffalo *(Bubalus bubalis,* 2*n* = 50) and swamp buffalo (*Bubalus carabanesis,* 2*n* = 48) [1] . The genetic difference is marked by a fusion of chromosomes 4 and 9 in swamp buffalo. The first cross between the two species produces fertile offspring, but fertility is reduced in subsequent crosses [2]. In comparison to cattle, buffalo metacentric chromosomes (five) are a fusion of two cattle acrocentric chromosomes and the other chromosomes conserve a high homology between species [3].

There are two water buffalo genomes sequenced [4, 5]. Both of them have the sequences available at NCBI platform in scaffolds. However, the sequences are not displayed in chromosomes and genes are not annotated (UMD_CASPUR_WB_2.0; http://www.ncbi.nlm.nih.gov/assembly/GCF_000471725.1/). In an effort to generate a reference set to aid polymorphism discovery and gene annotation of the buffalo genome, RNA from 30 different tissues was extracted and sequenced [6]. The lack of buffalo genomic data means that researchers need to refer to a “next of kin” species: cattle.

Cattle and buffalo species are in a close evolutionary relationship and the cattle genome is far better characterised than buffalo. An initial genome maps for buffaloes using cattle-derived markers and possible rearrangements were identified between species [7]. Positional candidate genes and physical mapping were generated giving a better understanding of buffalo genome structure.

More recently, a cattle SNP chip was applied to characterize buffalo genome. Genotyping 10 buffaloes with a 54 k cattle SNP chip (Illumina) and found that ~80 % of the SNPs were successfully genotyped, but only ~2 % (1,159) were segregating in the population [8]. This result indicates that genome sequences are conserved between the species but not necessarily the polymorphisms. The authors also identified that the SNPs genotyped are not equally distributed in the buffalo genome. There are some SNP-rich and some regions with poor SNP coverage, and therefore the cattle SNP chip does not offer an optimal coverage of buffalo genome. Genotyping 384 buffaloes using 777 k cattle SNP chip (Illumina) and showed that ~88 % of the SNPs were genotyped in buffaloes, but also only ~2 % (16,580) of the SNPs were segregating [9]. In a linkage disequilibrium study, these authors reported a mean value of r^2^ equal to 0.28 indicating that these SNP could be used for genomic selection and SNP association analyses. Studies that used cattle SNP chips did identify SNP associated with production and reproductive traits in buffaloes; using the 54 k cattle SNP chip [10] and a 777 k cattle SNP chip [11]. However, given that only ~2 % of the SNP in these cattle chips was segregating in buffaloes, a species specific SNP chip would be more informative. Importantly, SNPs present in the cattle chips that segregate in buffaloes are probably “old” polymorphisms, existent before the speciation event that separated cattle from buffaloes. Old polymorphisms might not be appropriate to study the result of artificial selection in dairy buffaloes. Based on this market necessity, a commercial buffalo SNP chip array was recently released (Axiom ® Buffalo Genotyping Array 90 K Affymetrix). The selection of SNP included in the chip array is based on buffalo sequencing data (Affymetrix), but SNP position and annotation to genes used the cattle genome as a reference (UMD3.1 assembly). Due to the fact that this is the most appropriate tool available, it was used in the present study.

The aim of the study was to identify SNPs, genomic regions and genes that affect production and reproductive traits. To this aim genome-wide association analyses and gene network predictions were carried. Gene network analyses aid the identification of genes that have pleiotropic effects and/or regulatory roles [12]. The genes identified might be candidates for future fine-mapping studies in search of causative mutations. The interpretation of results herein might also trigger genome structure, metabolism and physiology comparisons between species, supporting evolutionary studies.

## Methods

Animal ethics committee approval was not required for the present study. The data and samples used here were obtained from an existent databank of the Animal Science Department from São Paulo State University (Unesp), Jaboticabal-SP, Brazil. The department is responsible for the Milk-Recording Buffalo Program. The farmers gently contribute with phenotypes, pedigree information and samples of the animals.

### Data structure

Six production traits and four reproductive traits were targeted. The production traits were: milk production (MP), fat production (FP), protein production (PP), fat percentage (%F), protein percentage (%P) and somatic cell score (SCS). The reproductive traits were: age at first calving (AFC), calving interval (CI), open days (OD) and number of services per conception (NSC). The data analyzed was based on 11,530 lactations of 3,431 buffaloes, monthly recorded from 1995 to 2013. Murrah buffaloes were from 12 farms with 186 sires with registered daughters. The final pedigree archive had 14,346 animals. The structure of the data is presented in Table 1. The SCS doesn’t have normal distribution and it was transformed to the log scale, using the function: SCS = (log2(CCS/100.000)) + 3 [13].

**Table 1:** Structure of the data and descriptive statistics for milk (MP), fat (FP) and protein production (PP), fat (%F) and protein percentage (%P), somatic cell score (SCS), age at first calving (AFC), calving interval (CI), number of services per conception (NSC) and open days (OD).

Trait	Numbers	Mean	SD	CG
MP (kg)	11530	1864.14	448.67	298
FP (kg)	2890	110.34	35.41	96
PP (kg)	2890	84.73	22.89	96
%F	2890	7.02	1.09	96
%P	2890	4.24	0.72	96
SCS	2890	5.82	0.92	96
AFC(days)	3431	897.13	128.16	134
CI (days)	4729	407.54	32.33	106
NSC	4978	2^a^	-	186
OD (days)	6894	138.84	21.78	184

*SD* standard deviation, *CG* contemporary group

^a^Mode

Records were obtained apart from the fifth production day in milk. First five day of colostrum production were not considered. Only lactations longer than 90 days were used in the analyses. The cumulative milk production over 305 days (MP), fat production (FP) and protein production (PP) were calculated apart from the production in the milk-recording day. The %F, %P and SCS were the monthly record means per lactation. The age at first calving (AFC) was defined as the difference, in months, between the first calving and the birth of the buffalo. The calving interval (CI) was defined as the difference, in months, between consecutive calving events. The number of services per conception (NSC) is the number of artificial inseminations per conception for each buffalo. The open day (OD) is the difference, in days, between the calving and the subsequent conception. The contemporary group (CG) was formed by herd, year and calving season (October-March and April-September) for all the traits, except CI and by herd, year and birth season for CI. Each CG had at least four animals and trait records between ±3.5 standard-deviations of the group mean.

### Breeding value prediction

A repeatability animal model was used for the genetic analyses of all traits except AFC. For AFC, an animal model without repetition was used, because this trait can only be measured once. Variance components were estimated by Restricted Maximum Likelihood method (REML) using the Wombat software [14]. The model included the fixed effect of CG, age fitted as a co-variable (age of buffalo at calving, linear and quadratic) (except for AFC) and the random effects of additive genetic value, permanent environment (except for AFC) and residual. Fitted model scan be represented in matrix notation:

$$ y=X\beta +Za+Wp+e $$

or

$$ y=X\beta +Za+{e}_{\left(\mathrm{f}\mathrm{o}\mathrm{r}\ \mathrm{A}\mathrm{F}\mathrm{C}\right)} $$

Where β, a, p and e are the vectors of fixed effects, additive genetic value, permanent environment and residual, respectively; X , Z and W are the incidence matrix of fixed effects, additive genetic value and permanent environment. A brief report of the parameter estimates (heritability, genetic and phenotypic correlations) was included (Tables 2 and 3). The genetic values and their accuracies were obtained (Table 4), de-regressed as was proposed by [15] and used as pseudo-phenotypes for GWAS.

**Table 2:** Additive genetic variance (*σ*_*a*_^2^), permanent environment (*σ*_*pe*_^2^), residual (*σ*_*e*_^2^), and heritability (*h* ^2^) for the traits.

Trait^a^	*σ*_*a*_^2^	*σ*_*pe*_^2^	*σ*_*e*_^2^	*h* ^2^ ± *SD*
MP (kg)	46672.49	36946.85	103861.99	0.25 ± 0.03
FP (kg)	276.58	286.06	693.03	0.22 ± 0.03
PP (kg)	96.18	80.69	196.06	0.26 ± 0.03
F (%)	0.24	0.26	0.16	0.37 ± 0.04
P (%)	0.03	0.02	0.02	0.42 ± 0.04
SCS	0.58	0.64	2.17	0.17 ± 0.03
AFC (days)	348.15	-	1668.89	0.17 ± 0.02
CI (days)	275.132	399.013	4061.479	0.06 ± 0.01
NSC	0.03	0.13	0.22	0.08 ± 0.02
OD (days)	128.87	285.83	483.89	0.14 ± 0.03

^a^ *MP* milk production, *FP* fat production *PP* protein production (PP), %*F* fat percentage, %*P* protein percentage, *SCS* somatic cell score. *AFC* age at first calving, *CI* calving interval, *NSC* number of services per conception, *OD* open days

**Table 3:** Genetic correlation (above the diagonal) and phenotypic correlation (below the diagonal) among traits.

Traits^a^	MP	FP	PP	F	P	SCS	AFC	CI	NSC	OD
MP(kg)	**-**	0.74	0.93	−0.08	−0.11	−0.09	0.19	0.09	0.04	0.23
FP(kg)	0.86	**-**	0.76	0.64	0.28	−0.05	0.21	0.08	0.03	0.19
PP(kg)	0.97	0.84	**-**	0.16	0.29	−0.11	0.17	0.06	0.03	0.19
F(%)	−0.13	0.44	−0.17	**-**	0.49	−0.08	0.08	0.06	0.02	0.21
P(%)	−0.15	0.39	0.21	0.36	**-**	−0.10	0.04	0.03	0.02	0.18
SCS	−0.18	−0.23	−0.26	−0.08	−0.12	**-**	0.09	0.16	0.09	0.19
AFC(days)	0.26	0.29	0.31	0.15	0.19	0.26	**-**	0.39	0.26	0.22
CI(days)	0.14	0.31	0.29	0.19	0.22	0.22	0.56	**-**	0.41	0.27
NSC	0.11	0.12	0.13	0.09	0.09	0.16	0.31	0.29	**-**	0.33
OD(days)	0.29	0.36	0.28	0.21	0.18	0.29	0.36	0.37	0.41	**-**

^a^ *MP* milk production, *FP* fat production *PP* protein production (PP), %*F* fat percentage, %*P* protein percentage, *SCS* somatic cell score. *AFC* age at first calving, *CI* calving interval, *NSC* number of services per conception, *OD* open days

**Table 4:** Estimated genetic values means (GVM) and their accuracies for the traits studied for all the animals in the pedigree and only for the genotyped animals.

Traits^a^	Total		Accuracy		Genotyped animals		Accuracy	
	GVM	SD	Min	Max	GVM	SD	Min	Max
MP (kg)	169.63	89.32	0.64	0.99	232.78	71.28	0.86	0.99
FP (kg)	6.59	4.89	0.61	0.99	12.02	4.42	0.86	0.99
PP (kg)	5.28	4.32	0.61	0.99	7.89	2.86	0.86	0.99
F (%)	0.49	0.12	0.62	0.99	0.72	0.04	0.86	0.99
P (%)	0.14	0.07	0.61	0.99	0.52	0.03	0.86	0.99
SCS	0.35	0.11	0.62	0.99	0.65	0.03	0.86	0.99
AFC (dias)	3.78	4.89	0.61	0.99	−28.64	2.84	0.86	0.99
CI (dias)	1.28	2.18	0.59	0.99	−13.67	2.06	0.86	0.99
NSC	0.01	0.04	0.58	0.99	−0.08	0.01	0.85	0.99
OD (days)	3.89	2.93	0.62	0.99	−2.33	1.21	0.86	0.99

*SD* Standard deviation, *Min* Minimum, *Max* Maximum.

^a^ *MP* milk production, *FP* fat production *PP* protein production (PP), %*F* fat percentage, %*P* protein percentage, *SCS* somatic cell score. *AFC* age at first calving, *CI* calving interval, *NSC* number of services per conception, *OD* open days

### Genotyping and quality control

A total of 452 buffaloes (57 sires and 395 dams) were genotyped using the 90 K Axiom ® Buffalo Genotyping (Affymetrix). The animals genotyped were the ones with the best accuracies. The sires have at least 40 progenies and the dams at least three calvings and many of the dams are mothers of sires used in the herds. Initially, the SNP chip contained 92,826 markers. Sample quality control observed the call rate of 0.95 and above, and a heterozygozity of ± 3 standard-deviation of the mean. For SNPs quality control, thresholds were set for call rate (superior to 0.98), Hardy-Weinberg equilibrium (*P*-value test less than 10^−6^), and correlation between markers (if higher than 0.998 one SNP of the correlated pair was excluded). Also, coincident SNPs were eliminated. Minor allelic frequency was not used to discard markers. Some SNPs were genotyped twice when there was a probe in each strand for the same SNP. In the case of coincident SNP, the probe with the most animals genotyped was used. Markers present in Y chromosome and mitochondrial DNA were discarded. Markers in X chromosome were considered. The males have only one X chromosome, so they are always homozygous for the markers (0 or 2), females have two, so they were codified like the autosomes (0, 1 or 2). After the quality control, the number of SNPs retained for association analysis was 61,145.

### Genome-wide association study

In order to associate SNPs with the de-regressed breeding values (DEBV) of the studied traits, a mixed linear model was implemented using R software and GenABEL package [16]. The DEBV have information of the record of the animal genotyped as well as from their relatives. The reliability (source of information) varies among the animals, so the DEBV have heterogeneous variances corrected by the residual weights as proposed by [15]. The model implemented was:

$$ y=X\beta + \mu +\varepsilon $$

Where y is the vector of the DEBVs, X is the vector of the genotypes in the locus being tested, β is the fixed additive genetic value attributed to the locus, μ is the vector of the polygenic with normal distribution *μ* ~ *N*(0, *Gσ*_*u*_^2^) and *ε* is the vector of the residual error with normal distribution *ε* ~ *N*(0, *Iσ*_*e*_^2^).

The pedigree relationship matrix based on pedigree, G, describes the relation of the whole genome among the individuals, since it is estimated based on alleles identical by state (IBS) of the markers. The parameters *σ*_*u*_^2^ and *σ*_*e*_^2^ were estimated using Restricted Maximum Likelihood (REML) method for each SNP. The generalized least square (GLS) was used to estimate the β effects using the F test for the null hypothesis *H*_0_ : *β* = 0.

$$ y=x\beta +z\mu +e $$

Where *y* is the vector of the DEBVs, *x* is the design matrix, β is the vector of coefficients of the regression on recoded SNP genotypes; *z* is the incidence matrix for animal effects; *μ* ~ *N*(0, *Aσ*_*a*_^2^) is a vector of the polygenic animal effects and *e* ~ (0, *Iσ*_*e*_^2^) is the vector of residuals, in which *A* is an additive genetic relationship matrix of animals and *I* is an identity matrix, and *σ*_*a*_^2^ and *σ*_*e*_^2^ are the animal’s additive polygenic variance and residual error variance, respectively. SNP allele substitution fixed effects (*β*) and random background polygenic effects were evaluated in this model. Values in the design matrix, *x*, were coded as 0, 1, 2 for the SNP genotypes, representing the number of copies of the minor allele carried by the individual. The parameters and were estimated using Restricted Maximum Likelihood (REML) method for all SNP. The generalized least square (GLS) was used to estimate the β effects using the F test for the null hypothesis *H*_0_ : *β* = 0.

Subsequently, a Wald chi-square statistics was used to determine if the SNP was associated with the traits studied [17].

The percentage of the phenotypic variance (Vp) explained by each SNP was estimated according to the equation:

$$ \%{V}_p = 100\left(\frac{2p\left(1-p\right){\overset{,_{\prime }}{a}}^2}{\sigma_p^2}\right) $$

Where:

- α = allelic substitution effect
- p = allelic frequency for i^th^ observed SNP in the population
- *σ*_*p*_^2^ = Phenotypic variance estimate of the trait

### Multi-trait analysis, pleiotropic effects and gene network prediction

The association weight matrix (AWM) methodology [12] was adapted and used to build a gene network from GWAS output data. In the original description of AWM a key trait is selected to weight network predictions. In this study, the main idea was to identify genes that equally contribute to the variation observed in all ten traits studied, as these pleiotropic SNPs might be more useful for genetic evaluation in buffaloes. In this context, it was used the methodology described by [18]: instead of using SNPs *P*-values, *t*-values calculated served to ground gene network predictions, (t ≥ 2.80 ≈ *p* ≤ 0.05). These statistics determine the importance of the SNPs across the traits and are interpreted as a measure of pleiotropic effect. All the SNPs were used in the analysis regardless of their location. Normally in AWM, SNP-to-gene distances are considered prior to construction of gene networks. However, in this study, inclusion of all SNPs was preferred since genotyped SNPs were buffalo variants with locations annotated in the cattle genome (precise SNP-to-gene distances are actually unknown).

To identify significant SNP-SNP interactions we used the partial correlation and information theory (**PCIT**) algorithm [19]. Pairwise correlations across matrixrows were used to predict SNP-SNP interactions and hence build a genenetwork [12]. The SNP pairs significantly co-associated and with correlation higher than 0.85 had an edge (connection) established in the gene network, which was visualized using the Cytoscape software [20] and MCode App [21]. In the network, every SNP was a node and every significant interaction was an edge connecting two nodes. When a SNP was next to a gene (Variant Effect Predictor default), the gene ID was included in the network.

### Identification of SNP location and gene enrichment

Variant Effect Predictor (VEP) from Ensembl website was used to verify if the significant SNP was near a gene and determine the distance. Analyses were done using the cattle genome.

Gene ontology (GO) enrichment analyses were carried using Gorilla tool (http://cbl-gorilla.cs.technion.ac.il/) to aid interpretation of GWAS results. The top genes associated with the traits were compared to a genome-wide background gene list. Top genes were defined as genes with a SNP whose *P* < 0.001 (distance of the SNP to gene determined by VEP default). These GO enrichment analysis were carried for each trait separately.

## Results and Discussion

Single-trait-single-SNP GWAS was carried for six milk production and four reproductive traits in a population of dairy buffaloes. These GWAS in buffaloes used a specific SNP-chip designed for the species. Although the selection of SNP included in the chip array is based on buffalo sequencing data, SNP position and annotation to genes used the cattle genome as a reference (UMD3.1 assembly) because there is no public complete genome reference available for buffalos. The numbers of significant SNP were similar between traits, within significance thresholds (Table 5). They were also similar in number to those obtained by studies that used low density SNP chips in cattle [12, 22].

**Table 5:** Number of significant SNP at different p-values for each trait.

SNP *p*-value	Traits^a^									
	MP	%F	%P	FP	PP	SCS	AFC	CI	NSC	OD
0.05	2991	2853	2938	2960	2896	2916	2894	3016	2976	2927
0.01	585	621	593	613	567	620	605	587	576	601
0.001	54	72	53	55	52	65	68	55	57	54

^a^Traits: *MP* Milk production, %*F* fat percentage, %*P* protein percentage, *FP* fat production, *PP*  protein production, *SCS* somatic cell score, *AFC* age at first calving, *NSC* number of services per conception, *OD* open days

We described the data structure (Table 1) and have estimated heritabilities for the studied traits, which range from 0.06 to 0.42 (Table 2). The genetic correlations range from −0.11 to 0.93 (Table 3). These parameters are reported as they underpin GWAS results and gene network predictions. Data structure awareness is important context for GWAS common concerns: multiple testing and sample size limitations.

The SNPs that explained most of the phenotypic variance indicated regions of the genome that have an influence in the traits studied and indicate new candidate genes. Phenotypic variance percentage, positions and nearby genes were provided for these significant SNPs (Table 6). Significance of all the SNPs tested and percentages of phenotypic variance explained were reported as well (Additional file 1: Table S1).

**Table 6:** Genes near to the most significant SNPs (*p* < 0.0001) for ten production traits.

Trait^a^	SNP name	Chr	Position (bp)	%Vp	Within Gene	100 kb	<500 kb
MP	AX-85176853	1	80123930	0.46		*LOC100847171*	
						*BCL6*	
						*RTP2*	
						*SST*	
	AX-85092281	16	69184336	0.38		*PTGS2*	
						*LOC100295047*	
	AX-85110813	1	50360638	0.30	*ALCAM*		
	AX-85154407	14	73498202	0.27		*LOC101908004*	
	AX-85048498	3	14771362	0.22	*UBQLN4*		
%F	AX-85075989	6	64757051	0.14		*KCTD8*	
	AX-85070208	6	81132415	0.10		*LOC782855*	
						*LOC101904777*	
	AX-85172444	X	52208657	0.09	*LOC100294934*		
					*LOC100294888*		
	AX-85099016	16	21197965	0.07			*ESRRG*
							*TRNAY-AUA*
							*GPATCH2*
	AX-85061238	10	93617773	0.06	*GTF2A1*		
	AX-85056378	23	15403744	0.06	*LOC101902323*		
					*FOXP4*		
%P	AX-85104983	17	66753221	1.08		*LOC101903483*	
						*SART3*	
						*ISCU*	
						*CMKLR1*	
						*WSCD2*	
	AX-85050041	17	26092651	1.02	*-*	*-*	*-*
	AX-85081756	5	76365673	1.01		*MFNG*	
						*CARD10*	
						*USP18*	
FP	AX-85083515	16	57250501	0.77		*GPR52*	
						*LOC101908155*	
	AX-85044438	5	76008737	0.69		*TMPRSS6*	
						*LOC510185*	
						*C1QTNF6*	
						*SSTR3*	
						*LOC101906944*	
						*RAC2*	
						*MIR1835*	
						*CYTH4*	
	AX-85139912	23	50686128	0.61		*SERPINB6*	
						*SERPINB9*	
						*SERPINB1*	
						*WRNIP1*	
						*LOC101904132*	
						*MYLK4*	
	AX-85061966	9	15900553	0.59	*IMPG1*		
	AX-85105219	27	39157445	0.47	*LRRC3B*		
PP	AX-85174902	22	60177121	0.72	*LOC101905145*		
	AX-85051961	23	15787091	0.57	*CCND3*		
	AX-85110855	10	98773695	0.55			*FLRT2*
							*TRNAC-ACA*
							*LOC101906220*
							*LOC785091*
SCS	AX-85164456	16	71885776	0.004			*PROX1*
							*LOC100139281*
							*TRNAG-CCC*
							*RPS6KC1*
	AX-85093628	26	18748400	0.003		*HOGA1*	
						*MORN4*	
						*PI4K2A*	
						*AVPI1*	
						*MARVELD1*	
						*ZFYVE27*	
						*SFRP5*	
						*GOLGA7B*	
	AX-85136262	5	116846374	0.003	*ATXN10*		
	AX-85092482	7	103828217	0.002	*SLCO6A1*		
	AX-85149935	14	74769387	0.002		*RUNX1T1*	
AFC	AX-85049453	X	95335754	0.54	*LOC100299005*		
	AX-85061054	8	22838009	0.40		*LOC538589*	
						*IFNAD*	
						*IFNW1*	
						*LOC618947*	
						*IFN-TAU*	
						*LOC781948*	
						*LOC100335490*	
						*LOC101904956*	
						*LOC100847941*	
						*KLHL9*	
						*IFNAG*	
CI	AX-85069024	17	63515708	0.22	*TPCN1*		
	AX-85175568	10	30077602	0.21	SCG5		
	AX-85067656	26	49118646	0.20		*LOC101903522*	
						*MGMT*	
NSC	AX-85051327	12	71075601	0.04	*LOC100336232*		
	AX-85169432	1	69359581	0.02	*LOC100847188*		
	AX-85117598	23	48414155	0.02	*LY86*		
OD	AX-85113259	9	40931034	0.04	*FIG 4*		

^a^Traits: *MP* Milk production, %*F* fat percentage, %*P* protein percentage, *FP* fat production, *PP* protein production, *SCS* somatic cell score, *AFC* age at first calving, *NSC* number of services per conception, *OD* open days

Association analyses for the reproductive traits resulted in candidate genes for buffaloes that have known roles in reproductive physiology. For age at first calving (AFC), the gene coding for interferon-Tau, *IFN-TAU*, and other interferon genes were identified. Embryonic production of *IFN-TAU* is the primary signal for maternal recognition of pregnancy in buffaloes [23]. Another gene associated with AFC was LOC100299005 (*SELP*), a gene with up-regulated expression during inflammatory processes related to follicular atresia in cattle [24]. It is clear that modifications in the protein structure and/or in the expression levels of these genes could affect conception outcome and therefore impact on AFC [25, 26]. *SELP* gene, mapped to chromosome X, had the most significant SNP associated to AFC. The sexual chromosomes influence reproductive and andrological traits in cattle [27–29], among others traits, such as SCS and milk content in dairy cattle [30–33]. The results presented here add to this list and encourage the inclusion of sexual chromosomes in GWAS to avoid missing important information.

For calving interval (CI), a gene involved in spermatozoa acrosome reaction in humans was identified: *TPCN1* [34]. Spermatozoa acrosome reaction is necessary for fertilization and tends to be studied in the context of male fertility. The association of *TPCNI* with CI suggests an interesting thought: a gene related to male fertility might be more relevant to herd performance (in terms of CI) than genes related to female fertility. Increased conception rates after calving, and, as consequence, decreased CI may also reflect fertilization ability of bulls in the studied population. As a complex trait, CI may be influenced by several component traits linked to both male and female fertility, including spermatozoa quality andrological parameters [34].

For number of services per conception (NSC), the top gene found was LOC100336232 (*ABCC4*). This gene has its expression increased in the endometrium of pregnant cows [36] and pigs [37] and seems to be important to support pregnancy since it acts on prostaglandin efflux from cells [36]. Prostaglandin has a variety of roles in reproduction being responsible for maternal recognition of the pregnancy and conceptus implantation, processes that closely related to NSC. Moreover, in a whole genome re-sequencing of Hanwoo cattle, *ABCC4* was identified as the gene with the biggest number of non-synonymous SNPs, splice-site variants, and coding indels [37]. *ABCC4* may be a useful source of variation to be studied in buffaloes and cattle. In Angus cattle, the *ABCC4* expression was significantly correlated with residual feed intake (RFI) [39], being up-regulated in high RFI animals. In Nelore cattle, a CNV within intron 22 of *ABCC4* was correlated with marbling score [40]. The emerging hypothesis is that *ABCC4* acts in basic metabolic pathways and is highly polymorphic with potential effect in a variety of phenotypes (i.e. reproduction, meat quality, etc.).

Gene ontology enrichment analyses were also performed using GOrilla to compare the top genes associated with the traits (*P* < 0.001) with a genome-wide background gene list. For AFC and CI, many processes involving neural development and activity were listed (GO terms: GO:0048814 and GO:0021836 for AFC and GO:0050807, GO:0045666, GO:00501962, GO:0050769, GO:0051960, GO:0031290, GO:0051960, GO:0031290, GO:0021819 for CI). There were other genes expressed in the central nervous system that were associated to puberty in female cattle [12, 22, 41]. The role of these genes in reproduction may be due to the neuronal activity in the hypothalamus-pituitary axis, responsible for initiating the hormone cascade that is a trigger for puberty followed by the initiation of the estrous cycle in females [42]. It is reasonable to assume that genes involved with pubertal development and maintenance of estrous cycle could be associated with AFC and CI.

Regarding %F and FP, four genes related to the carbohydrate metabolism (*KCTD8*, *FOXO4*, *SSTR3*, *LOC782855)* and one gene related to lipid metabolism (*ESRRG*) were identified. The *KCTD8* gene interacts with genes that act in the insulin secretion and glucagon liberation pathways, participating in the glucose absorption [43]. *FOXO4* gene down-regulates gluconeogenesis and up-regulates glycolysis [44]. *SSTR3* inhibits the activity of Glucose-dependent insulinotropic polypeptide’s function in intestine, promoting the accumulation of glucose and fat [45]. *LOC782855* (*RPS26*) was related to diabetes in humans [46]. The association of *ESRRG* to fat production in the present GWAS could be expected, since this gene regulates other adipogenic genes [47]. In cattle, *ESRRG* was also considered a key regulator of puberty in a multi-trait analyses that included fat deposition traits [12]. Most of the top genes associated with fat percentage and fat production integrate the carbohydrate metabolism and not the lipid metabolism as in cattle [48, 49]. This fact could suggest some differences between buffalo and cattle fat production in milk. On average, buffaloes have higher contents of milk fat than cattle. In buffaloes the percentage of milk fat ranges from 6.7 % to 12.0 % [11, 50–52], while in cattle it ranges from 3.1 % to 4.5 % [53, 54]. The difference in milk fat might be explained by a more efficient acetate metabolism to produce lipids in buffaloes, as the results suggest. In comparison to cattle and under same high fibber diet, buffaloes have a higher average daily gain [55]. It means that buffaloes have a better capacity to digest fibber content in rumen. Fibber fermentation generates acetate, a fat precursor [56]. This characteristic might generate a bigger contribution of genes related to acetic acid metabolism in the fat production traits in buffaloes, differentiating considerably cattle and buffalo metabolism for fat milk content [56].

SNP associations with milk protein production suggested *CCND3* as a candidate gene in buffaloes. This gene has a role in alveolar development in the mammary gland, in cooperation with prolactin [57]. Variances in the biological activity of protein coded by *CCND3* may affect the structure and/or physiology of the alveolus. Milk production is a function of blood circulation in the mammary alveolus. Other genes, whose physiological activity is within the alveolus, were already correlated with milk content in buffaloes [58]. Now, *CCND3* has been added to the list with specific implications for milk protein content.

Gene ontology enrichment analyses were also carried for production traits. For the somatic cells score (SCS), the biological process of regulation of lymphocyte migration was significant (GO:2000401). This immunological metabolic process is correlated with SCS because this trait is used as an indirect measure of mastitis which severely diminishes milk production. Buffaloes with a more efficient immune system (better variants for genes that regulate lymphocyte migration) might do better in avoiding the disease.

Buffalo and cattle chromosomes have an extensive similarity and 84 % of cattle markers were successfully used in buffaloes [7]. However, despite good examples of putative candidate genes reported herein (mainly obtained due to the similarities), many genes that were associated to milk production traits have no known role in milk production. Some of these genes are described as having a role in basic metabolism and many are not characterized at all. The associations presented here open the door to study these genes in the context of milk production. It also reinforces that basic research to characterize and identify the function of the genes is still necessary, especially in buffaloes. According to [59], there are only 493 annotated genes in buffalo. The regular number of genes in mammalian genomes is around 20,000, so the discrepancy is evident. Moreover, rearrangements and inversions in the cattle homologue chromosomes complicate the annotation of buffalo genes [60]. A species specific genome reference for buffalo is needed.

**Fig. 1:**
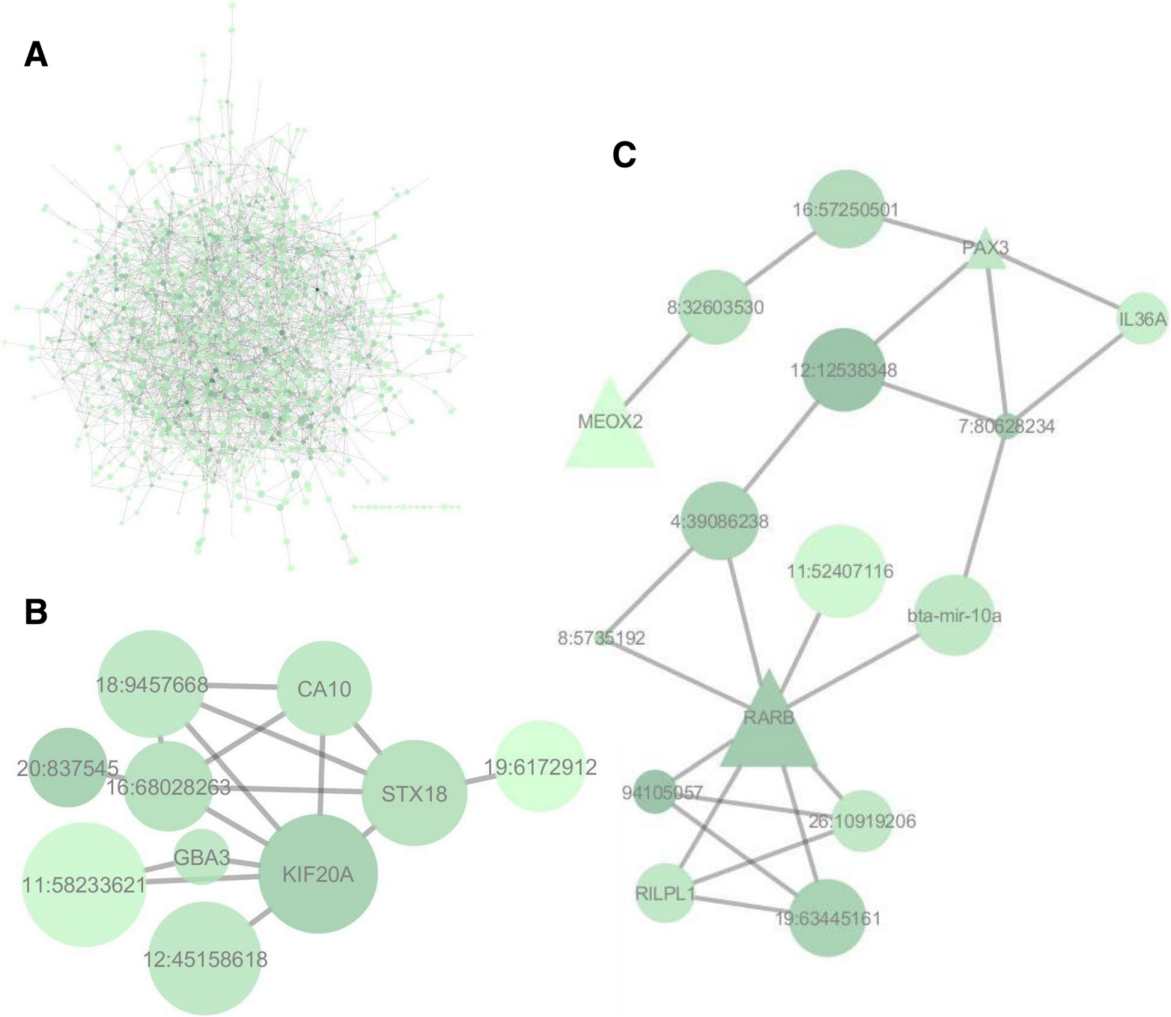
**a** - Association weight matrix gene network. **a**) Entire gene network formed by 1,068 nodes connected by 3,307 predicted interactions (edges). **b** – Highest interconnection region in the network whose seed (square) is *CA10* gene (MCODE App). **c** - Subset of the co-association network showing the best duo of transcription factors: *RARB* and *ATF1*

The identification of genes with pleiotropic effects could contribute to the genetic evaluation of many traits. An example of a gene with important pleiotropic effects is *PLAG1* in cattle [61]. To identify genes with a pleiotropic effect and regulatory role in buffaloes, we predicted a gene network from the ten studied traits. The gene network was visualized using Cytoscape software. Data from 1,723 SNPs were used in network predictions, selected SNP were associated with the majority of traits. Of these, a total of 608 SNPs were identified to be close or within a gene or a known transcript. The SNPs, that didn’t have a gene close to them, remained on the network as nodes named after their chromosome position. The final gene network had 1068 nodes and 3307 edges. The nodes interactions varied from 23 to 1 with an average of 3.9 interactions per node (Fig. 1a).

A SNP located in *C14H8orf34* gene was a central node of the network, having 23 predicted interactions. Information about this gene is scarce; however some indications of its function and pleiotropic effect might be discussed. SNPs in this gene were associated, in humans, with “fasting serum aspartate aminotransferase” and “urinary free epinephrine excretion per day” [62]. Some considerations might be done concerning these phenotypes. Aspartate aminotransferase is a test carried to check for liver damage [63]. The liver has more than 400 functions and participates in the general metabolism. A gene with a liver function would be a logical candidate for pleiotropic or regulatory roles. The association of *C14H8orf34* with epinephrine production may also support a pleiotropy claim. Epinephrine is a hormone and a neurotransmitter synthesized in the adrenal glands that acts via many pathways to accelerate metabolism under stress situations [64].

With the aim to find high interconnected regions, termed clusters, further analyses were carried using MCode App (Cystoscape). The first cluster had *CA10* as its seed (Fig. 1b). The function of *CA10* is the inter-conversion between carbon dioxide and bicarbonate, with essential physiological function in many tissues [65]. In humans, SNPs in *CA10* were related to menarche, weight and body mass index [66, 67]. The association of this gene with growth and reproductive traits reinforces the wide effect and supports the findings herein. In this context, *CA10* might be a regulator of fat metabolism and reproductive development in buffaloes.

Transcription factor (TF) genes, a total of 28 in the network, also had their clusters mined for regulatory information. Genes that work as TF are important in terms of pleiotropy effect since they can guide transcription and interact with many other genes. The TF with highest number of predicted interactions was *RARB,* a retinoic acid receptor important for cell growth and differentiation [68]. *RARB* is expressed in many tissues from liver and intestine [69] to sperm [70] in cattle. The gene seems also to be a very important for bovine mammary gland cell viability [68]. Studies of cell cycle and apoptotic events in mammary glands [68] suggests that the role *RARB* in its development is expressive. Considering the traits herein analysed, six out of ten traits analysed are related to the udder and its physiology, so the finding of a TF that has a crucial role in its development as a central regulator in the network is plausible (Fig. 1c).

The TF gene with the second largest number of connections in the network was *ATF1*. This gene regulates other genes involved in growth and survival and was associated with angiogenesis in the mammary gland [71]. Milk is derived from blood due to difference of pressure in the alveolus. A suitable explanation is that the better vascularised the alveolus are, the bigger is the milk and contents production, resulting in a suitable explanation. This gene was also indicated as a key TF for meat quality [72] (Fig. 1c).

The *RARB* gene has eight interactions, three of them are with known genes (*RILPL1*, *bta-mir-10a*, *PRKCA*) and *ATF1* gene has six interactions, four of them are with known genes (*ABCA13*, *TBC1D19*, *MAGI2, CUL5*). It is curious to verify that some of these genes also have a variety of roles in the metabolism and are also candidates to have pleiotropic effects.

A genome wide association study with milk production was done with the buffalo SNP chip in Mediterranean buffaloes [73] with 78,137 SNPs considered in the analyses. However, the SNPs reported to be highly associated with the trait [73] are not the same to the ones found in the present study. Some of the SNPs weren’t included in the present analyses and some didn’t have significance. The divergent results could be explained in many ways: different genetic composition of the breeds (Mediterranean x Murrah), selection pressure in the population (expected to be higher in the Italian population), inbreeding (higher in Brazilian population), SNPs segregating and analyzed in both studies (78,137 SNPs in Mediterranean x 61,144 SNPs in Murrah), methodologies for estimation of the SNP effects and etc.

GWAS studies were done for reproductive and production traits in China [10], and using partially the same animals of the present study [11] and a low and a high density bovine SNP, respectively. The genes found were not the same as the above discussed neither. There are many differences in the markers used in these studies. They worked with 935 SNPs [10] and 15,745 SNPs [11] that are cattle variants and are also segregating in buffaloes. These markers should exist before the differentiation of the species. In the present work, the density of the SNPs is much higher (61,144 markers) and the selection of them based on buffaloes data. Differences in SNPs used may explain the contrasting results. The bovine SNP chip does not cover the buffalo genome with the same efficiency, even if the species are close in evolution terms. The use of cross-species SNP chips might not be as informative as initially proposed [59]. Even if the DNA sequences are similar, the functional variants may not be, as suggested before [8] and [10]. The interesting candidate genes discussed herein resulted from significant SNP and are largely supported by the literature in terms of its biological function. These promising GWAS results emphasize the importance of selecting SNP that are species specific.

Dairy cattle GWAS of varying breeds and traits were further evidence for species differences when compared to results herein [30, 32, 33, 49, 74–79]. Only one SNP (rs41610147) was associated with three fertility traits in Danish Holstein cattle (female fertility index, interval from calving to first insemination, days from first to last insemination in heifers) [76] and was located in an association region in buffaloes. The SNP (rs41610147) in cattle is 398 bp far from the second most significant SNP associated with NSC in buffaloes. These SNPs may be indicating the same causative mutation or major gene associated to reproductive physiology in both species. It is possible to conclude that despite high genome homology between buffalo and cattle, the contribution and influence of genes and variants to studied traits is mostly different. Candidate genes that might be buffalo-specific could be explained by the presence of underpinning causative mutations that are not found in cattle. Some examples of divergent time of genes between the species were discussed before [59].

Comparing different breeds of the same species already results in differences regarding associated genes and the proportion of phenotypic variance that they explain [80, 81]. These differences might be explained by epistatic effects, selection pressure, different environment, recombination rate, effective population size, allelic frequencies differences, genome coverage by the SNP chip etc. Logically, if differences are found between breeds of the same species, comparing different species can only result in stronger contrast.

Species-specific technologies are important and needs to be further developed. Particularly, for the buffalo species, the lack of a publicly available complete and annotated genome complicates the advance and development of new methodologies for genetic evaluation for the specie.

The genes identified in this study are candidates for fine-mapping with the aim to find putative causative mutations. The incorporation of this information in a low density SNP chip is informative and auxiliary to genetic evaluation, with cost-benefit for producers. The identification of causative mutations reduces the need for tag SNP (in linkage disequilibrium with causative mutations), promotes higher accuracy for genomic breeding values, which can persist over generations and permits a higher transferability across breeds [82]. The results presented expand our knowledge and indicate regions for possible genes not yet annotated in buffaloes, but potentially important. It also serves as basis for further functional genes studies.

## Conclusion

The present article is a genome wide association and gene network analyses in buffaloes using a SNP chip specifically developed for the species. Putative genes for production and reproductive traits were found and these are candidates for searching causative mutations. Comparative analyses between cattle and buffaloes support that although the genome sequence is similar, the variants between them are different. Evidence that species-specific technology should be developed for buffaloes was presented discussed herein.

## Additional file

Additional file 1: Table S1. P-values, allelic substitution effect and percentage of phenotypic genetic variance of the SNPs used in the GWAS for the ten traits in dairy buffaloes. (XLSX 30.7 mb)

## Abbreviations

%F: Fat percentage
%P: Protein percentage
AFC: Age at first calving
AWM: Association weight matrix
CG: Contemporary group
CI: Calving interval
CNV: Copy number variation
DEBV: De-regressed breeding values
FP: Fat production
GLS: Generalized least square
GO: Gene ontology
GWAS: Genome-wide association study
MP: Milk production
NCBI: National Center for Biotechnology Information
NSC: Number of services per conception
OD: Open days
PCIT: Partial correlation and information theory
PP: Protein production
REML: Restricted Maximum Likelihood method
RFI: Residual feed intake
SCS: Somatic cell score
SNP: Single nucleotide polymorphism
TF: Transcription factor
VEP: Variant Effect Predictor
Vp: Phenotypic variance
